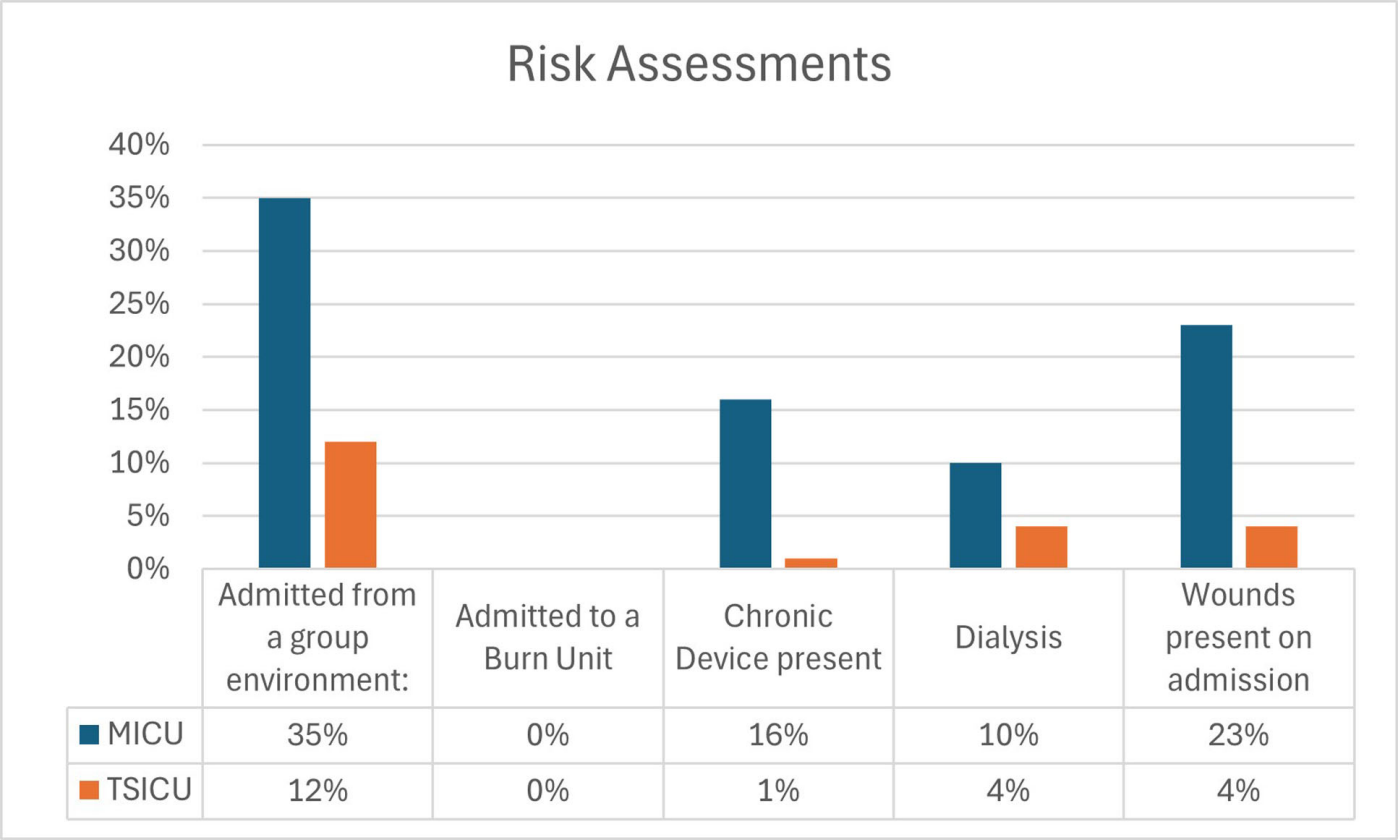# 245 Evaluating the Effect of Educational Interventions On Inappropriate Urinalysis with Reflex to Culture Orders in Catheterized Patients

**DOI:** 10.1017/ash.2026.10617

**Published:** 2026-06-23

**Authors:** Jennifer Sanguinet, Gerard Marshall, Mary Jo Foreman, Justin Peruchini, Rian De Guzman, Jeffrey Murawsky

**Affiliations:** 1 Sunrise Hospital & Medical Center; 2 Sunrise Hospital and Medical Center; 3 HCA Far West Division

## Abstract

**Background:** Candida auris (C. auris) is a pathogen that causes high morbidity in patients due to its resistance to antimicrobials. Endemic patient colonization poses an infection risk that may be mitigated by no touch disinfection systems through interruption of the infection chain reservoir step. We evaluated the efficacy of continuous dry hydrogen peroxide (DHP) exposure on C. auris hospital onset colonization to evaluate the interruption of the transmission post hospital admission. **Methods:** The study was conducted in two intensive care units within a large tertiary-care center from October 2024 to January 2025. DHP-emitting systems were installed in the Medical Intensive Care Unit (MICU) HVAC system. Trauma Surgical Intensive Care Unit (TSICU) had no DHP installed. Admission and post admission testing was completed on all patients present in both units. All non-positive patients were tested twice per week and removed from testing if positive. Presence of C. auris was determined by polymerase chain reaction (PCR) from composite swabs from axillae and groins. Patients were screened for C. auris risk factors according to a standard risk assessment (Figure 1). The study goal was to determine if there was a statistically significant difference (PC. auris days to critical care days for TSICU and MICU patients with hospital-onset C. auris using the N-1 Chi-squared test (proportion comparison). **Results:** Risk assessments for MICU (268) and TSICU (297) were evaluated. The MICU population showed a higher risk for C. auris in each category compared to TSICU (Figure 1). MICU had double the dialysis, triple the admissions from group environment, and 5 times more wounds present on admission. The length of stay in TSICU was about 2.3 days less than MICU. There is a statistically significant difference (PC. auris days to critical care days for TSICU and MICU. No adverse effects were reported by patients, visitors, or personnel associated with the DHP systems. **Conclusion:** This study demonstrates that DHP was effective in reducing the proportion of hospital-onset C. auris colonization when comparing the MICU (DHP installed) to the TSICU (without DHP). This effect was observed despite the MICU having a longer average length of stay and a higher percentage of patients with known risk factors for C. auris. The findings support DHP as a valuable no-touch disinfection method. Figure 1: Risk assessment results by location, MICU or TSICU

**Figure:**